# Computer Simulation of the Movement of Listeria Bacteria

**DOI:** 10.1371/journal.pbio.0020436

**Published:** 2004-11-30

Each of the cells in our body is a complex machine. Within each cell, thousands of proteins and other molecules interact to produce the highly organized cellular events that are needed for life. However, there is no cellular line manager telling the individual proteins where to go and what to do. It's rather like an ant colony. No one tells the individual ants how to build a nest. They just do it. Similarly, no one tells the individual actin molecules, for example, to get together and polymerize to form the cytoskeleton that is essential for cell movement and other cellular processes. It just happens.

Ant colonies and actin polymerization are both examples of emergent behavior. Broadly defined, emergent behavior is when a collection of individuals interact without central control to produce results that are not explicitly programmed. Scientists are only just beginning to understand how complex behaviors of this type can emerge from a myriad of individual interactions, many of which are well-defined experimentally.

Jonathan Alberts and Garrett Odell have turned to an unlikely ally—the bacterium Listeria monocytogenes—in their study of the complexities of cellular actin polymerization. This rod-shaped microbe, which lurks in well-ripened brie and other unpasteurized cheeses, can cause the sometimes fatal disease listeriosis, particularly in young babies or people with weakened immune systems.

When Listeria microbes in food reach a person's gut, they penetrate the cells lining the gut, reproduce, and then spread from cell to cell without ever exposing themselves to the extracellular environment. In this way, they avoid the host's immune system. One particular protein produced by Listeria is central to this sneaky intracellular lifestyle: ActA. Expression of ActA allows the microbes to hijack the machinery in the host cell that controls the growth of actin networks. Some of the cellular actin, instead of forming the cytoskeleton of the cell, polymerizes around the bacterium, forming a dense “comet tail” of actin that generates a force to move the bacterium around the cell and push it into neighboring cells. This clandestine use of our cellular machinery for actin polymerization is far simpler to understand than the elaborate use our cells normally make of it, so the study of Listeria propulsion provides a scientific stepping-stone to understanding the involvement of the actin cytoskeleton in normal cell movements.

Alberts and Odell have used established data on the biochemical and mechanical details of actin polymerization and the physiological concentrations of the proteins involved in the process to build a computer simulation of how an actin network assembles around a moving, rod-shaped bacterium. Their “in silico” reconstitution, which considers the behavior of individual actin filaments and requires a cluster of 80 computer processors to be run for several days at a time, produces realistic bacterial motion in terms of speed and persistence, and models the actin tail shape. The model also reproduces smaller scale emergent behavior. Real Listeria cells do not move smoothly. Instead, the microbes move jerkily, with runs interspersed with pauses. The simulation faithfully reproduces this type of movement and offers a mechanistic explanation for it.

The approach described by Alberts and Odell can now be used to investigate many other, more complex cell mechanics problems, such as how the cellular movements involved in cell division are achieved. With the availability of detailed biological information and powerful computers, we may at last start to solve the mystery of how interactions among maybe 100,000 gene products can produce the organized cellular processes that cell biologists have been watching under the microscope for years.

